# Individual-level analysis of MRI T2 relaxometry in mild traumatic brain injury: Possible indications of brain inflammation

**DOI:** 10.1016/j.nicl.2024.103647

**Published:** 2024-07-22

**Authors:** Mayan J. Bedggood, Christi A. Essex, Alice Theadom, Samantha J. Holdsworth, Richard L.M. Faull, Mangor Pedersen

**Affiliations:** a Department of Psychology and Neuroscience & TBI Network, Auckland University of Technology (AUT), New Zealand; b Mātai Medical Research Institute, Gisborne, New Zealand; c Department of Anatomy and Medical Imaging, Faculty of Medical and Health Sciences & Centre for Brain Research, The University of Auckland, New Zealand

**Keywords:** mTBI, Concussion, Magnetic resonance imaging, Neuroinflammation, Neuroimaging

## Abstract

Mild traumatic brain injury (mTBI), often called concussion, is a prevalent condition that can have significant implications for people’s health, functioning and well-being. Current clinical practice relies on self-reported symptoms to guide decision-making regarding return to sport, employment, and education. Unfortunately, reliance on subjective evaluations may fail to accurately reflect the resolution of neuropathology, exposing individuals with mTBI to an increased risk of further head trauma. No objective technique currently exists to assess the microstructural alterations to brain tissue which characterise mTBI. MRI-based T2 relaxation is a quantitative imaging technique that is susceptible to detecting fluid properties in the brain and is hypothesised to indicate neuroinflammation. This study aimed to investigate the potential of individual-level T2 relaxometry to evaluate cellular damage from mTBI. 20 male participants with acute sports-related mTBI (within 14 days post-injury) and 44 healthy controls were recruited for this study. Each mTBI participant’s voxel-wise T2 relaxometry map was analysed against healthy control averages using a voxel-wise z-test with false discovery rate correction. Five participants were re-scanned after clinical recovery and results were compared to their acute T2 relaxometry maps to assess reduction in potential neuroinflammation. T2 relaxation times were significantly increased in 19/20 (95 %) mTBI participants compared to healthy controls, in regions including the hippocampus, frontal cortex, parietal cortex, insula, cingulate cortex and cerebellum. Results suggest the presence of increased cerebral fluid in individuals with mTBI. Longitudinal results indicated a reduction in T2 relaxation for all five participants, indicating a possible resolution over time. This research highlights the potential of individual-level T2 relaxometry MRI as a non-invasive method for assessing subtle brain pathology in mTBI. Identifying and monitoring changes in the fluid content in the brain could aid in predicting recovery and developing individualised treatment plans for individuals with mTBI. Future research should validate this measure with other markers of inflammation (e.g. from blood biomarkers) to test whether T2-relaxometry is related to subtle brain inflammation in mTBI. In addition, future research should utilise larger control groups to establish normative ranges and compute robust z-score analyses.

## Highlights

• This study shows how T2 relaxometry MRI can quantify structural brain changes of acute mild traumatic brain injury (mTBI).
• We observed increased T2 relaxometry in specific brain regions of 95% of mTBI participants.
• After recovery, there was a decrease in T2 relaxometry.
• T2 relaxometry may reflect subtle neuroinflammation and help develop personalized treatment for mTBI.

## 1 Introduction

Approximately 69 million people worldwide experience a mild traumatic brain injury (mTBI) each year (Oris et al., 2023). Whilst in many cases, people recover well, others can experience chronic, disabling impacts that affect their daily functioning (McInnes et al., 2017, Verboon et al., 2021). mTBI occurs when a person experiences a strong force on the head that causes the brain to move within the skull. The predominant mechanism of injury is a sudden impact, rotational force or rapid deceleration or acceleration of the brain. The primary injury in mTBI is from the immediate mechanical damage to the brain, resulting in focal and diffuse damage (Alam et al., 2020, Verboon et al., 2021). Following this primary injury, a cascade of secondary injuries can occur. These include inflammation, excitotoxicity, metabolic disturbances, vascular damage, and blood–brain barrier disruption (Kim et al., 2023, Slavoaca et al., 2020, Soltani et al., 2020).

The term ‘mild TBI’ may not correspond to the personal experience of many people, as many of those with an mTBI experience long-lasting symptoms that persist beyond the two to four weeks ‘standard’ recovery time (Kara et al., 2020). The symptoms that accompany the secondary injuries can, if left untreated, last for weeks, months or even years (Slavoaca et al., 2020). Common symptoms of mTBI include headaches, difficulty concentrating, sleep difficulties, a foggy feeling, vestibular disorders, confusion, slowed reaction times, nausea, changes in vision, sensitivity to light, and irritability (Di Battista et al., 2020, Markovic et al., 2021, Verboon et al., 2021). In the short term, these symptoms can have severe negative impacts on the lives of patients’. In the longer term, mTBI has been associated with reduced work productivity and increased risk of psychiatric and neurodegenerative diseases (Theadom et al., 2017, Wang et al., 2021).

Neuroinflammation is one of the primary drivers behind the secondary injuries associated with brain injuries (Markovic et al., 2021, Piao et al., 2013). The initial tissue damage triggers the activation and recruitment of immune cells by facilitating the production of cytokines and chemokines (Kim et al., 2023). The effect of this inflammatory response is to limit the spread of injury in the brain and to restore homeostasis. Microglia play a key role in responding to inflammatory events by identifying structural abnormalities and working to isolate damaged regions of the brain to prevent the spread of injury. Shortly after the injury, microglia release proinflammatory cytokines, a vital and adaptive part of neuronal preservation (Alam et al., 2020) Neuroinflammation can be beneficial in the acute phase following brain injury, promoting repair of the damaged tissue, possibly inducing neurogenesis and reducing the risk of infection (Monsour et al., 2022). However, when this inflammation occurs in excess, it can contribute to the loss of neurons and death of brain tissue, resulting in a secondary injury cascade (Corps et al., 2015, da Luz Scheffer et al., 2020, Kim et al., 2023, Markovic et al., 2021, Mee-Inta et al., 2019).

T2 relaxometry is a novel MRI method that is used to assess microstructural tissue alterations that can accompany different neurological diseases and conditions. This type of MRI provides an indicator of intracellular and extracellular injury (Pell et al., 2004), such as in epilepsy (Adel et al., 2023, Winston et al., 2017). The most important property to consider that impacts T2 relaxation time is the water content of the tissue. This water can consist of free water molecules, which are smaller and have a faster spin frequency and a longer T2 relaxation time. Or, the water content can be bound with larger macromolecules with a spin frequency comparable to the Larmour frequency with shorter T2 relaxation times. In regular, healthy tissue, these two types of water exist in equilibrium, but in many pathological conditions (e.g. inflammation), the bound water is released and the free water increases. This creates an inefficient medium for T2 relaxation; therefore, T2 relaxation time increases as tissue water content increases and can indicate pathology (Cheng et al., 2012, Ghugre et al., 2011, Liu et al., 2018).

In mTBI patients, brain regions with higher T2 relaxometry signals could reflect accumulated fluid caused by the secondary damage to the brain. Previous research has applied quantitative T2 MRI in other brain disorders, such as epilepsy; however, few studies have used this technique with mTBI. Pedersen et al. (2020) identified possible brain inflammation in a professional Australian Rules football player who had incurred multiple mTBIs, demonstrating the potential promise of this technique. This study found that abnormally elevated T2 relaxometry trended toward baseline at each additional MRI throughout the recovery period, but had not yet resolved at the final follow-up. Furthermore, T2 relaxometry is a marker of TBI in animal studies and mouse models of mTBI indicate that T2 relaxometry can be a valuable tool for identifying acute neurostructural perturbations post-mTBI (Yang et al., 2015).

Standard clinical neuroimaging post-mTBI is often negative, with no clinically significant findings (Mayer et al., 2015). This can perpetuate the notion that the injury did not result in neuronal pathology despite the patient presenting as symptomatic. Ultimately, there is a need for an advanced method of quantitative MRI that can detect subtle changes in the brain acutely after mTBI. Advanced T2 MRI methods may uncover individual abnormalities that are hypothesised to be indicative of possible neuroinflammation despite clinically normal radiology findings. In this context, T2 relaxometry could indicate areas of fluid accumulation, and possibly transient neuroinflammation, post-mTBI and validate subjective symptom reports or indicate pathology despite a lack of subjective symptoms. Acute T2 relaxometry measures could be utilised to detect injury and predict recovery outcomes from mTBI based on the quantified fluid in the brain upon acute presentation.

The current study comprises a series of 20 acute mTBI cases individually compared to a control group. This technique will enable a detailed investigation of potential brain abnormalities on an individual level, enabling any findings to be considered in the context of the patient’s clinical presentation. Deepening our understanding of the underlying pathology of mTBI could contribute to individualised treatment plans and improved patient recovery outcomes.

## 2 Methods

### 2.1 Participants and procedure

This study consists of 20 male sports players (*M* = 21.6 years old [16–32], *SD* = 4.76) with acute (≤14 days) sports-related mTBI. Injuries were sustained from participation in rugby union (n = 13), football (n = 2), hockey (n = 1), futsal (n = 1), gymnastics (n = 1), surfing (n = 1), and jiu-jitsu (n = 1). The population template for comparison was derived from 44 male controls that had not suffered an mTBI in the last 12 months and had no lingering symptoms from an mTBI (*M* = 23.3 years old [16–30], *SD* = 4.38). The age of individual mTBI participants and the average age of controls may vary slightly between the 20 individual statistical tests, as our hypothesis is to conduct individual-specific analyses rather than estimate between-group differences. The difference between the mean age of mTBI and control groups was not statistically significantly (*p* = 0.14). See Table 1 for a summary of participant demographics and injury details. Participants in the mTBI cohort were recruited through Axis Sports Concussion Clinics in Auckland, New Zealand and via community pathways (e.g. physiotherapists, word-of-mouth, digital and print advertisements). The control group were recruited through print and social media advertisements, and word-of-mouth. Brain Injury Screening Tool (BIST) questionnaires were completed to determine symptom severity and clinical recovery (Theadom et al., 2021). Five mTBI participants were re-scanned after clinical recovery. All research was conducted in accordance with the Declaration of Helsinki. Ethics approval was obtained from the Health and Disability Ethics Committee (HDEC – 2022 EXP 11078), New Zealand and all participants provided written informed consent prior to data collection.

**Table 1:** Participant demographic and injury details.

**Case**	**Post-injury MRI (days)**	**Prior mTBI’s**	**Recovery (days)**	**Symptom Reports post-injury**	**BIST** **(/160)**	**Injury details**	**Health issues**
1	5	1	20	Headaches, tiredness, emotional, feeling like he was “in a dream”	140	Rugby − knee to head	None
2	12	1	31	Headaches, nausea, fatigue	12	Hockey − ball to face (left eye)	Unknown
3	13	0	27	None reported	78	Football − in goal, slipped and fell backwards hitting head on ground	Unknown
4	13	1	26	Visual disturbances (seeing “stars”), unsteady, headaches, sensitivity to light	18	Rugby − head collision	Unknown
5	10	1	56	Impaired balance, disoriented, feeling “not well”, visual disturbances	61	Gymnastics − fell off bar and onto back, two days later fell again and hit top of head	ADHD, anxiety, depression
6	5	0	26	Vomited, ringing in ears, dizziness, headaches, neck pain, light sensitivity, difficulty focusing	42	Rugby − lifted and dumped onto his neck in a tackle causing cervical hyperflexion	None
7	6	0	47	Impaired balance, dizziness, headaches, sensitivity to light and noise, difficulty concentrating, irritable	13	Rugby − kicked in the back of the head	Unknown
8	10	2	27	None reported	6	Rugby − knee to the head	Unknown
9	13	?	18	Double vision, fogginess, slowed thinking, indecisiveness	56	Rugby − head hit ground	Unknown
10	11	10	?	None reported	54	Surfing − hit head on board	Unknown
11	13	4	24	Dizziness, dazed, headaches, neck pain, sensitivity to light, fogginess, mild cognitive symptoms	52	Rugby – head-to-head collision	Unknown
12	12	0	23	Loss of consciousness, “zoning out”, headaches/pressure in the head, difficulty concentrating	13	Rugby − left side of head hit ground and right side of head hit player’s knee/elbow	Unknown
13	13	?	17	Dazed, pressure in head, neck pain, fogginess, reduced cognitive sharpness	79	Rugby − head to knee and then elbow to face	None
14	13	1	20	None reported	2	Football − ball kicked into face and fell to ground	None
15	6	?	18	Ataxia, confusion	22	Rugby − struck on right side of face with elbow	Unknown
16	12	4	117	Impaired balance, blurry vision, “pins and needles” in hands, dizziness, headaches	117	Rugby − knee to back of head	History of migraines
17	5	2	66	None reported	0	Rugby − shoulder hit head	None
18	13	0	26	None reported	34	Rugby − knee to head	Unknown
19	8	0	32	None reported	28	Futsal − elbow to face	Unknown
20	13	2	31	None reported	69	Jiu-jitsu − knee to right side of head	None

### 2.2 Magnetic resonance imaging

All magnetic resonance images were acquired using a 3 T Siemens MAGNETOM Vida fit scanner (Siemens Healthcare, Erlangen, Germany) located at the Centre for Advanced Magnetic Resonance Imaging (CAMRI) at The University of Auckland, New Zealand, using a 20-channel head coil. A T2 mapping sequence was used to investigate the anatomical T2 relaxometry. T2 maps were acquired using an 8 echo Carr-Purcell-Meiboom-Gill (CPMG) sequence (TEs = 28.9, 57.8, 86.7, 115.6, 144.5, 173.4, 202.3 and 231.2 ms; TR = 6s; slice thickness = 2.0 mm; voxel size = 2.0 x 2.0 x 2.0 mm; matrix size = 112 x 128 x 63; flip angle (FA) = 180°; base resolution = 128; phase resolution = 100 %; phase field of view (FOV) = 87.5 %). Total T2 mapping acquisition time was ∼ 12:02 min. Note that accelerate mapping methods, e.g., GRAPPATINI (Hilbert et al., 2018), can greatly reduce acquisition time which makes T2 mapping more clinically feasible. See Supplementary Materials 1, for mean and coefficient of variation for the T2-maps across all subjects. T1-weighted anatomical images were collected for quality control purposes. The T1 weighted images were acquired using a magnetisation-prepared rapid gradient echo (MPRAGE) sequence (TR = 1.9 s; TE = 2.5 ms; TI = 979 ms; FA = 9°; slice thickness = 0.9 mm; voxel size = 0.4 x 0.4 x 0.9 mm; matrix size = 192 x 512 x 512; phase FOV = 100 %). Total T1-weighted acquisition time was ∼ 4:31 min.

A radiologist reviewed clinically relevant MRI images from each participant to check for clinically significant abnormalities that might require further attention.

### 2.3 Data processing and statistical analysis

All MRI images were received in *DICOM* format, converted to *NIfTI* format, and arranged according to the *Brain Imaging Data Structure (BIDS)* (Gorgolewski et al., 2016)*.* Image quality assurance checking was conducted in the *MR View* toolbox of *MRtrix*3 (Tournier et al., 2019) by two investigators in the study (MJB, MP) using the participants’ T1 weighted image as the underlay and their T2 map as the overlay to check for artifacts or abnormalities caused by scanning or processing. T2 maps were skull stripped using the *bet* function in FSL (Smith, 2002) before normalising each image to the MNI standard space by registering them to a MNI152 2.0 x 2.0 x 2.0 mm template image using *FSL FLIRT* (Jenkinson et al., 2002). For each subject, the third T2 echo volume (86.7 ms) was extracted to generate a group average brain image and binary grey matter, white matter, and cerebrospinal fluid masks. Lastly, we removed the first volume, using the offset as a fitting parameter to the T2 relaxation data (Milford et al., 2015) and a monoexponential function at each voxel was fitted across all eight echo-times using *qMRLAb* (Karakuzu et al., 2020) in *Matlab R2022* to calculate the T2 relaxation time for each participant.

Z-tests were used to quantify differences between individual mTBI subjects and controls. Before statistical analysis, each image was smoothed using a 6 mm full-width-half-maximum (FWHM) kernel. All voxels residing within the grey matter mask were considered for final analysis. Given that the main statistical assumption of a z-test is that the underlying data distribution is Gaussian with a mean of 0 and standard deviation of 1, we used a Rank-Based Inverse Normal Transformation (Blom, 1954) to normalise the data. All T2-relaxometry voxels for each image were ranked and transformed into a Gaussian shape with a distribution mean of 0 and a standard deviation of 1. Z-score maps were obtained by subtracting voxel values between individual mTBI maps and the mean of controls divided by the standard deviation of the controls. This is a similar individual-based statistical approach to previous studies (Jolly et al., 2021, Mito et al., 2023, Pedersen et al., 2020).

**Fig. 1:**
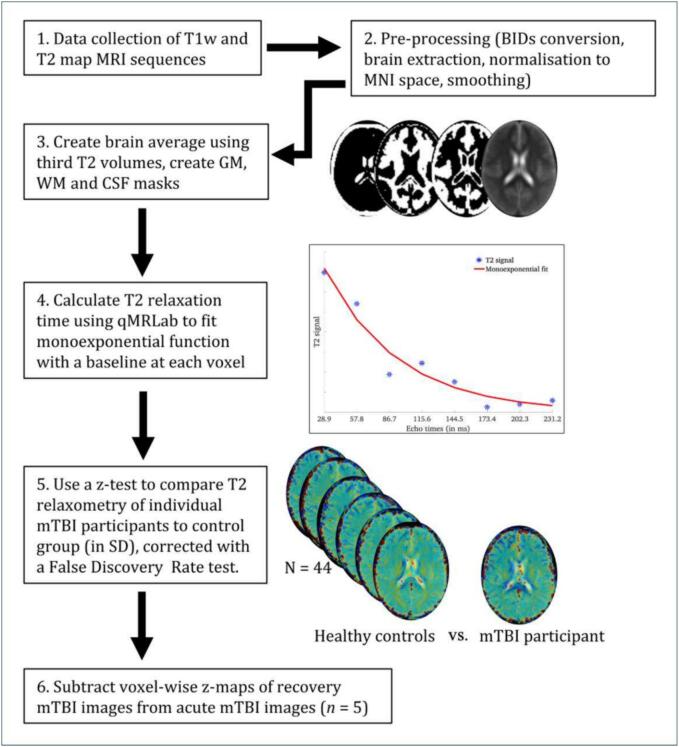
Methodology for MRI data processing and analysis.

We conducted a one-tailed z-test, interpreting positive values only, as the biology of increased T2 relaxometry is well understood, i.e., increased water properties in tissue (Cheng et al., 2012, Ghugre et al., 2011, Liu et al., 2018). In contrast, the interpretation of decreased T2 relaxometry is less well understood. False Discovery Rate (FDR – Benjamini & Hochberg, 1995) was used to correct for multiple comparisons in the z-score maps using a threshold of *q* < 0.05. This method obtained z-values and relaxometry times (in ms) for each FDR-based significant cluster, individually for each mTBI participant. Lastly, to determine if the significant T2 relaxometry clusters had resolved with clinical recovery, we subtracted the acute voxel-wise z-maps of the five participants with recovery MRI re-scans from the voxel-wise z-maps of their recovery MRI scans. See Fig. 1 for a schematic overview of our processing and analysis pipeline.

## 3 Results

### 3.1 Summary of acute scan findings

**Fig. 2:**
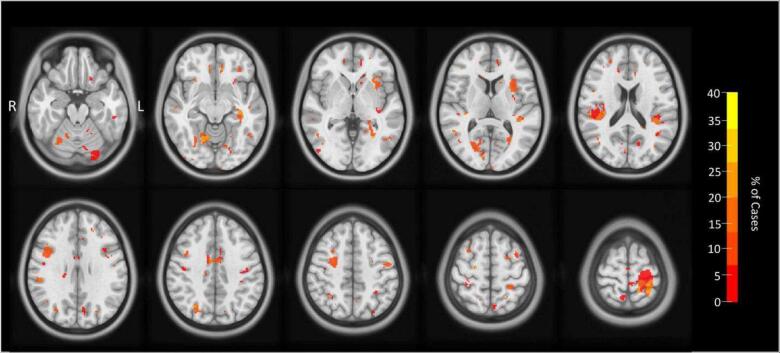
Common regions of significant T2 relaxometry.

The quantitative T2 analysis indicated regions with significantly higher T2 relaxometry times in 19/20 mTBI participants (95 %), detailed in Table 2. While several significant voxel clusters, such as the cingulate cortex, superior parietal cortex and insula, were shared across multiple participants (see Fig. 2), most of the findings were individual to each case study. A radiologist analysed each participant's MRI scans, and no clinically significant findings were reported.

**Table 2:** Individual results showing voxel clusters in mTBI with significantly higher T2 relaxometry times compared to controls.

**Case**	**Region(s)**	**Raw T2 Relaxometry (ms)**	**Z-score compared to controls (SD)**
**1**	L superior parietal L lateral superior frontal L medial superior frontal L orbitofrontal cortex	146.50 2.69e + 06* 74.18 79.78	8.40 4.08 4.38 6.50
**2**	L superior parietal L anterior insula R superior frontal	63.67 63.72 72.10	9.39 18.65 6.91
**3**	R somatosensory cortex R intraparietal sulcus	76.10 51.54	8.66 15.69
**4**	L anterior insula L posterior cerebellum	68.26 66.95	12.12 7.04
**5**	R anterior parietal	55.86	12.83
**6**	L hippocampus L lateral occipital	95.22 71.83	5.00 9.33
**7**	L superior parietal R intraparietal sulcus R temporoparietal junction R superior cerebellum	73.15e + 04* 72.34 65.49 89.35	7.26 14.39 3.40 8.72
**8**	Cingulate cortex L inferior supramarginal gyrus	73.47 76.10	8.27 16.31
**9**	L superior parietal R posterior insula	65.66 70.28	8.81 5.31
**10**	L superior parietal R inferior parietal R superior parietal	54.89 65.95 70.83	15.92 14.48 8.46
**11**	L parahippocampal cortex L orbitofrontal cortex R anterior occipital R orbitofrontal cortex	76.46 6.50 79.23 74.74	10.37 4.83 10.22 4.34
**12**	R superior cerebellum R superior temporal sulcus	79.46 69.54	13.15 10.23
**13**	L anterior temporal	125.00	4.70
**14**	L inferior supramarginal gyrus L anterior occipital L hippocampus R premotor cortex	70.15 57.68 274.06 87.64	25.44 13.83 8.38 19.60
**15**	R inferior parietal R posterior parietal R superior cerebellum R medial prefrontal cortex	70.64 66.73 104.69 83.92	12.33 19.85 5.78 8.13
**16**	R sensorimotor cortex	74.02	14.86
**17**	Cingulate cortex L superior frontal L posterior cerebellum	99.04 259.39 1.61e + 08*	11.08 6.05 5.37
**18**	L superior frontal	303.65	5.28
**19**	None	N/A	N/A
**20**	L anterior cingulate cortex L medial frontal cortex L sensorimotor cortex L precuneus L temporoparietal junction R anterior cingulate cortex R superior parietal	66.32 121.10 79.65 38.98 100.25 73.53 22.05	17.51 5.68 4.57 5.49 5.60 15.48 4.89

*As these peak relaxometry values are very high, they could be coming from voxels outside the brain, in the cerebrospinal fluid. However, parts of the clusters appear to be situated within the brain, so they have been included in the analyses.

### 3.2 Case series of recovery re-scan findings

Recovery re-scans indicated either a full reduction (i.e. in all significant acute regions) or a partial reduction (i.e. for some areas but not others) in T2 relaxometry for all five re-scanned participants. The remainder of the Results section will provide comprehensive details about the five case studies that had recovery re-scans, with information regarding their injury, symptoms and specific T2 relaxometry results for specific areas of their brains. A detailed description of individual acute scan results for the remaining participants can be found in the Supplementary Materials 2.

#### 3.2.1 Case 1

**Fig. 3:**
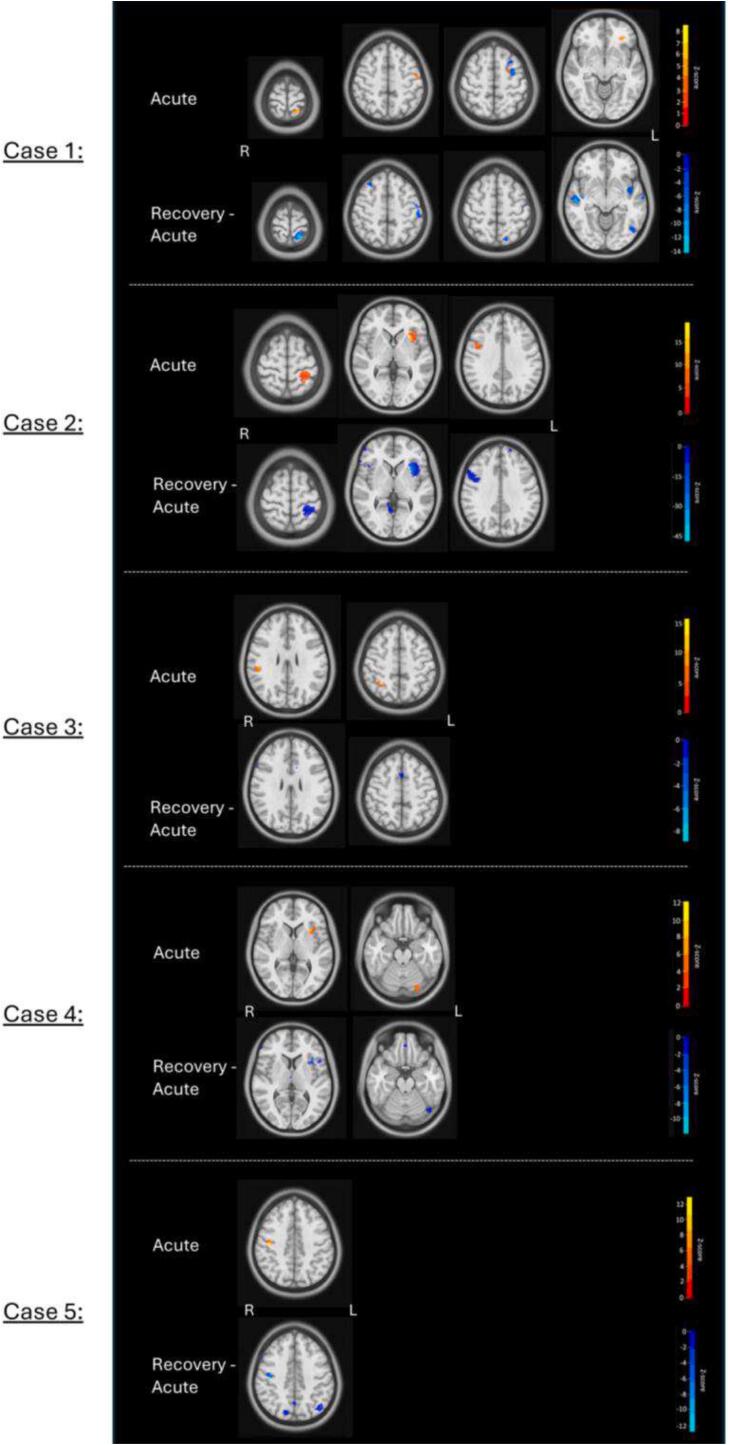
T2 Relaxometry acutely following mTBI (top) and at recovery (bottom) (in SD) for cases 1–5.

Case study one suffered an mTBI as a result of a knee to the head during rugby. He experienced headaches, tiredness, abnormal emotional expression and feeling like he was in a dream. These symptoms largely settled at the clinical assessment four days post-injury, with only mild headaches and tiredness being experienced. Brain Injury Screening Tool (BIST) scores were low, with no individual statements obtaining abnormally high scores and an initial total score of 12/160. This patient has experienced one other mTBI in their life, during primary school. Recovery time for the current mTBI is recorded as 20 days. Four significant voxel clusters are apparent when assessing the T2 relaxometry MR images (see Fig. 3). There is a significant cluster in the left superior parietal region, with relaxometry times up to 8.40 SD higher than the controls (peak relaxometry value = 146.5 ms). Two significant clusters are found in the left superior frontal region, the first more laterally with relaxometry times up to 4.08 SD higher than controls (peak relaxometry value = 2.7e + 06 ms). As the peak relaxometry value is very high, it could indicate that this peak value is coming from a voxel situated outside the brain, in the cerebrospinal fluid. However, some parts of the cluster appear to be located within the superior frontal region of the brain. The second left superior frontal region is more medial, with relaxometry times up to 4.38 SD higher than controls (peak relaxometry value = 74.2 ms). Lastly, there is a significant cluster in the left orbitofrontal cortex, with relaxometry times up to 6.50 SD higher than controls (relaxometry value = 79.8 ms). After recovery, re-scans indicated that T2 relaxometry was reduced by 14.09 SD in the left superior parietal region, 2.01 SD in the left lateral superior frontal region, and 4.52 SD in the left orbitofrontal cortex. No significant reduction was found for the left medial superior frontal region cluster.

#### 3.2.2 Case 2

Case study two suffered an mTBI when a ball hit his left eye during a hockey game. He experienced developing headaches and nausea after the match and reported mild headaches and fatigue at his clinical assessment nine days post-injury. The patient’s BIST scores were low, with no symptom domains or individual statements obtaining abnormally high scores and an initial total score of 6/160. Recovery time for this injury is recorded as 31 days. Furthermore, he suffered one previous mTBI in 2022. Three significant voxel clusters were identified when analysing the T2 relaxometry images (see Fig. 3). The first is in the left superior parietal region, with relaxometry times up to 9.39 SD higher than controls (peak relaxometry value = 63.7 ms). There are also significant clusters in the left anterior insula, with relaxometry times up to 18.65 SD higher than controls (peak relaxometry value = 63.7 ms) and in the right superior frontal region, with relaxometry times up to 6.91 SD higher than controls (peak relaxometry value = 72.1 ms). After clinical recovery, re-scans indicated that T2-relaxometry was reduced by 8.66 SD in the left superior parietal region, 40.88 SD in the left anterior insula, and 6.15 SD in the right superior frontal region.

#### 3.2.3 Case 3

Case study three suffered an mTBI when he collided with another player during a football game. The patient did not report any specific symptoms during his clinical assessment 11 days post-injury. The patient’s BIST scores were low with no symptom domains or individual statements obtaining abnormally high scores and an initial total score of 13/160. The patient reported no previous mTBIs and the recovery time for the current mTBI was recorded as 27 days. Two significant voxel clusters are identified when analysing the T2 relaxometry images (see Fig. 3). The first is in the right somatosensory cortex in the parietal lobe, with relaxometry times up to 8.66 SD above controls (peak relaxometry value = 76.1 ms) and the second is in the right intraparietal sulcus, with relaxometry times up to 15.69 SD higher than controls (peak relaxometry value = 51.5 ms). After clinical recovery, re-scans indicated that T2-relaxometry was reduced by 1.54 SD in the right somatosensory cortex and by 0.06 SD in the right intraparietal sulcus.

#### 3.2.4 Case 4

Case study four had a head-to-head collision with a teammate during a rugby game and suffered an mTBI as a result. At the time of injury, the patient reports “seeing stars” and being unsteady, followed by headaches and sensitivity to light. Symptoms resolved within a few days and were exacerbated approximately nine days post-injury during Jujitsu training. At his clinical assessment, 12 days post-injury, the patient reports headaches and fatigue. BIST scores were low, with no symptom domains or individual statements obtaining abnormally high scores and an initial total score of 22/160. The patient has reported one previous mTBI, in 2021. Recovery time for the current mTBI is recorded as 26 days. When analysing T2 relaxometry MRI data for this patient, two significant clusters of voxels were discovered (see Fig. 3). The first is in the left anterior insula, with relaxometry times up to 12.12 SD higher than controls (peak relaxometry value = 68.3 ms) and the second is in the left posterior cerebellum, with relaxometry times up to 7.04 SD above controls (peak relaxometry value = 67.0 ms). After clinical recovery, re-scans indicated that T2-relaxometry was reduced by 11.22 SD in the left anterior insula, but no reduction was found in the left posterior cerebellum.

#### 3.2.5 Case 5

Case study five sustained an mTBI during gymnastics training when he fell off a high bar. The patient reports stumbling when returning to his feet but not experiencing any other symptoms at the time of injury. The following day, he reports feeling disoriented and unwell. Two days after the first injury, the patient again falls on his head during gymnastics training. This time, the patient reports seeing flashes of colour and blurred vision. No additional symptoms are reported during his clinical assessment seven days post-injury. BIST scores were low, with no symptom domains or individual statements obtaining abnormally high scores and an initial total score of 34/160. Recovery time for this injury is recorded as 56 days, and this patient has suffered one previous mTBI in approximately 2017/2018. When analysing the T2 relaxometry images, one significant cluster of voxels was found in the right anterior parietal lobe (see Fig. 3), with relaxometry times of up to 12.83 SD above controls (peak relaxometry value = 55.9 ms). After clinical recovery, re-scans indicated that T2-relaxometry was reduced by 12.68 SD in this region.

## 3 Discussion

### 3.1 Individual-level brain abnormalities in acute mTBI

Group-level statistical analyses dominate clinical neuroscience, and while they have utility in enabling reliable comparisons between groups, they may not detect brain abnormalities unique to individuals. Our study compared each mTBI patient to the average of controls, enabling a comprehensive analysis of significant voxel clusters for specific individuals. We found that 19/20 mTBI patients had significantly higher T2 relaxometry times than the control group (see Fig. 3). Regions with significantly higher T2 relaxometry are summarised in Table 2 and include the left hippocampus, left superior parietal cortex, left superior frontal cortex, left orbitofrontal cortex, left supramarginal gyrus, left anterior insula, left posterior cerebellum, right temporoparietal junction, right superior cerebellum, right medial prefrontal cortex, right somatosensory cortex, and the cingulate cortex. Given T2 relaxometry’s sensitivity to quantify water properties, we hypothesise that our findings could indicate signs of subtle neuroinflammation during acute stages of mTBI. These findings are corroborated by our recent T2-relaxometry group analysis (Bedggood et al., 2024). Here, we compared 40 mTBI and controls, and the results indicated that whole-brain average T2-relaxometry was significantly increased in the mTBI group compared to controls – which further validates our current finding that 95 % of participants in this case series had increased T2-relaxometry compared to controls. However, the group analysis results indicated that increased T2-relaxometry were widespread, particularly in superior cortical regions of the brain, which highlights the potential differences between individual and group imaging markers in clinical populations.

A radiologist reviewed MRI scans from all participants, and none were deemed to have any findings that would warrant clinical follow-up. This indicates that the patients in this study may have potentially pathological brain abnormalities despite no findings on hospital-based MRI protocols. Of note, the radiologist's report for case 18 did indicate a structural brain change in the left superior frontal gyrus (see Supplementary Materials 2). This region was not deemed clinically significant by the radiologist and likely unrelated to the current brain injury. Still, this region spatially aligned with our findings of a significant T2 relaxometry voxel cluster in the left superior frontal lobe and further supports our hypothesis that T2 relaxometry can identify structural brain abnormalities. This further demonstrates the utility of T2 relaxometry for uncovering subtle abnormalities.

### 3.2 Common regions of increased T2 relaxometry across mTBI individuals

As well as individual variation in T2 relaxometry times, we observed a range of significant voxel clusters that multiple participants had in common, affecting up to 40 % of mTBI cases (Fig. 2). These regions include the cingulate cortex, anterior insula, cerebellum and superior parietal cortex. Regions with increased T2 relaxometry times across multiple participants could indicate that specific brain regions are more susceptible to acute brain changes following mTBI. If there were regions that appear more vulnerable, these regions could serve as potential biomarkers for injury and predictive indicators for recovery from mTBI for future players. For example, fluid accumulation in specific brain regions acutely following injury may be linked to poorer recovery. Therefore, early identification of this pathology could facilitate individualising treatment plans (e.g., a more conservative return to sport) and improve recovery outcomes.

### 3.3 Recovered mTBI participants show reduced T2 relaxometry

For all five participants with longitudinal data available, MRI recovery re-scans suggest that increased T2 relaxometry present in the acute scans is reduced, at least partially, once they are clinically recovered (see Fig. 3). These recovery re-scans provide valuable evidence to support the idea that the fluid accumulation found on acute scans was linked to the mTBI, as it seems to resolve as the participant recovers. This evidence aligns with a previous case report suggesting that brain-specific T2 relaxometry reduces after clinical recovery (Pedersen et al., 2020). This finding raises the question of whether mTBI is an acute or chronic injury with regard to brain pathology. Conducting recovery re-scans with a larger sample size would enable conclusions regarding whether this pattern is generalisable to the mTBI population. If our findings are replicated at a larger scale, they could be utilised as an objective measure of recovery from mTBI.

### 3.4 Clinical implications of increased T2-relaxometry

Conventional MRI images are generally qualitative. Without a quantitative metric to interpret the signal intensities (independent of scanner hardware and sequences), comparing MRI images longitudinally or across subjects is challenging. While the conventional method provides good tissue contrast, the signal intensity can only be interpreted qualitatively. Consequently, interpreting the resulting images relies on selecting an appropriate sequence and a sound understanding of the signal contrast relative to the pathophysiology. By not depending on this subjective process and to obtain quantitative information, the contributions of different contrast mechanisms need to be extracted (Cheng et al., 2012). Quantitative measures can isolate the contributions of individual MR contrast mechanisms (i.e. T1, T2 and T2*). The relaxation time is sensitive to water content, iron levels and tissue structure. It measures the biophysical parameters by decoupling the contrast mechanisms contributing to the signal.

Uncovering regions with increased T2-relaxometry and possible acute inflammation following mTBI could affect the individual’s recovery and return to play. For example, gaining a deeper understanding of brain abnormalities post-injury could help clinicians individualise treatment plans instead of basing the plan on group averages, self-reports, or generic guidelines for recovery. To base treatment decisions on personal data means tailoring the plan for what would benefit the individual − for example, increasing or decreasing the recommended stand-down period before returning to play. Therefore, the chance of being too lenient in the approach to rest and recovery is reduced. Furthermore, knowing whether someone’s brain was showing signs of pathology could instigate a need for altering the recommended physical activity, as it has been linked to inflammation (da Luz Scheffer et al., 2020, Godinho et al., 2021, Kreber and Griesbach, 2016, Mee-Inta et al., 2019), or it could entail incorporating other methods for reducing inflammation (e.g. anti-inflammatory medications – Bergold, 2016) that would not have otherwise been suggested. Overall, understanding brain pathology on an individual level and subsequently customising treatment plans could result in a more efficient and safer return to play for athletes with an mTBI.

### 3.5 Limitations and future directions

The current study has several limitations to consider when discussing the results. Firstly, while all MRI scans were conducted during a 14-day window in the ‘acute’ stage following mTBI, there was inter-participant variability in the exact timing and injury severity. Minor timing and severity differences could be a potential explanatory variable to consider for differences in neuroinflammation. Furthermore, the current study only included male participants which could reduce transferability to wider mTBI populations. If both sexes were to be included, separating them into different groups and comparing them would be sensible. This would require a larger sample size and reduce the feasibility of the study. With the selection of only males, it maintains homogeneity and allows for a substantial sample size to be collected for individual analysis. Larger future studies could include females too and look at group differences. Although our simulations suggest that 44 controls are sufficient for a reliable z-test (see Supplementary Materials 3), future research would benefit from utilising larger control groups in order to establish normative ranges, or predictive machine learning approaches, in mTBI. However, we can envisage challenges conducting this type of single-subject analysis in patients with gross brain abnormalities due to lack of an equivalent control brain and potential image distortions during MNI normalisation process. Lastly, while the BIST was utilised to measure injury and clinical recovery, our future research aims to include additional clinical and behavioural data as well as more standardised clinical and functional measures to encapsulate a more comprehensive picture of participants’ clinical presentation.

Although longitudinal research is challenging (especially as MRI studies are a large commitment for the participants), it would be beneficial for future research to re-scan more mTBI participants after recovery. This way, T2 relaxometry times, and therefore sites of possible neuroinflammation, could be compared in the ‘injured’ and the ‘recovered’ brains of participants to see if significant clusters in the acute scans normalise with recovery. The possibility that increased T2-relaxometry indicates subtle inflammation in the brain is, at this stage, still uncertain and requires further validation. Future research should validate this measure with other markers of inflammation, for example IL-10, a pro-inflammatory marker that also promotes microglial recovery in rat models of mild TBI injury (Maiti et al., 2019).

## 4 Conclusion

We observed that nearly all mTBI participants had evidence of elevated brain T2 relaxometry after an mTBI. We hypothesise that these findings could indicate possible neuroinflammation and enable a deeper understanding of their injuries' pathology. However, this remains uncertain and needs to be validated with further research. Furthermore, comparing the recovery MRI scans of five participants to their acute scans indicated that T2 relaxometry reduces at recovery. This suggests a possible transient nature to the brain pathophysiology and provides a possible objective imaging technique for reliably judging recovery following mTBI. Quantitative T2 relaxometry methods provide individual, detailed maps of potential brain abnormalities and may offer new insights into a patient’s clinical presentation. Understanding the role that this increase in T2-relaxometry, and possible inflammation, plays acutely post-mTBI could have implications for individualising treatment approaches and, therefore, improving recovery outcomes for mTBI patients.

## CRediT authorship contribution statement

**Mayan J. Bedggood:** Writing – review & editing, Writing – original draft, Visualization, Project administration, Methodology, Formal analysis, Data curation, Conceptualization. **Christi A. Essex:** Writing – review & editing, Project administration, Methodology, Investigation, Data curation. **Alice Theadom:** Writing – review & editing, Writing – original draft, Supervision, Resources, Methodology, Investigation, Funding acquisition, Conceptualization. **Samantha J. Holdsworth:** Writing – review & editing, Methodology, Investigation, Funding acquisition, Conceptualization. **Richard L.M. Faull:** Writing – review & editing, Methodology, Investigation, Funding acquisition, Conceptualization. **Mangor Pedersen:** Writing – review & editing, Writing – original draft, Supervision, Methodology, Investigation, Funding acquisition, Formal analysis, Data curation, Conceptualization.

## Declaration of competing interest

The authors declare that they have no known competing financial interests or personal relationships that could have appeared to influence the work reported in this paper.

## Data Availability

De-identified data will be made available upon reasonable request.
