# Supplementary material for: Individual-level analysis of MRI T2 relaxometry in mild traumatic brain injury: Possible indications of brain inflammation
Source: Neuroimage Clin. 2024 Jul 22;43:103647. doi: 10.1016/j.nicl.2024.103647 (PMC11663787; doi:10.1016/j.nicl.2024.103647)

**Supplementary Material 1**

We averaged the 8 T2-map echoes for all subjects (mTBI and controls). We calculated the mean (top row) and coefficient of variation, in % (middle row), and coefficient of variation overlaid with the grey matter mask used in our study. Most variability across subjects was seen proximate to the edges of the brain and ventricles, but these voxels were not included in our study due to our grey matter mask.


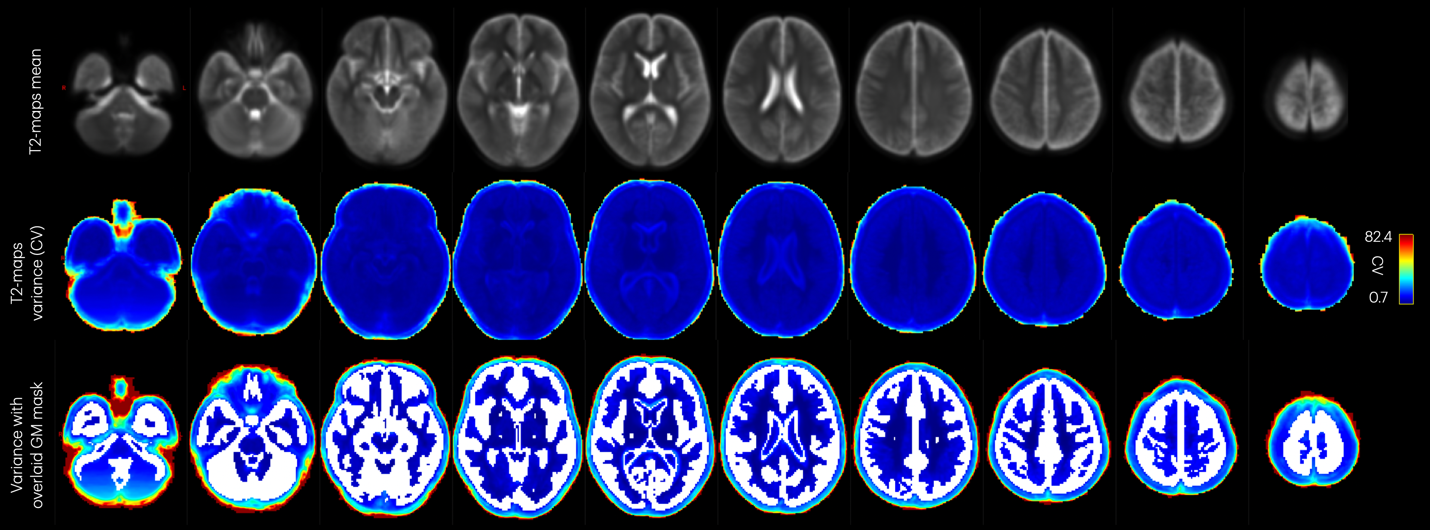


**Supplementary Material 2**

**Case 6**

Case six suffered an mTBI resulting from a rugby tackle whereby he was dropped on his neck. Clinical reports indicate some vomiting, dizziness and ringing in the ears immediately post-injury, as well as neck pain, headaches, light sensitivity and difficulty focusing four days post-injury. BIST scores are high and indicate a 10/10 rating for the statements “I don’t like bright lights” and “I don’t like loud noises”, as well as a 9/10 rating for the statement “I have trouble with my eyesight (vision)”, an 8/10 score for “I forget things” and a total initial BIST score was 140/160. This is the first mTBI that this patient has experienced, and recovery time was recorded as 26 days. Two significant findings are discovered after examination of T2 relaxometry MR images. Firstly, there is a significant cluster of voxels in the left hippocampal region, with relaxometry times up to 5.00 SD higher than that of the control group (peak relaxometry value = 95.2 ms). Secondly, there is a significant cluster in the left occipital lobe, with relaxometry times up to 9.33 SD higher than controls (relaxometry value = 71.8 ms).

**Case 7**

Case study seven received a kick to the head during a rugby tackle and suffered an mTBI as a consequence. Directly after the incident, the patient complained of feeling dizzy and off-balance and at his clinical assessment four days post-injury, he reported headaches, sensitivity to light and noise, as well as difficulty concentrating and some increased irritability. BIST scores indicate a 6/10 rating for the statement “I don’t like bright lights”, a 7/10 for the statement “I don’t like loud noises”, a 6/10 for the statement “It takes me longer to think” and a 7/10 rating for the statement “I have trouble concentrating”. His total initial score was 78/160. The patient has not experienced any previous mTBIs and recovery time is recorded as 47 days. Four significant voxel clusters were discovered when assessing the patient’s T2 relaxometry images. Firstly, there is a significant cluster in the left superior parietal region, with relaxometry times up to 7.26 SD above the control average (peak relaxometry value = 731,456.0 ms). Again, as this peak relaxometry value is very high, it may be coming from a voxel that is situated outside the brain, in the CSF. However, a portion of the significant cluster appears to be situated within the superior parietal region of the brain. Secondly, there is a significant cluster in the right intraparietal sulcus, with relaxometry times up to 14.39 SD higher than controls (peak relaxometry value = 72.3 ms). Next, there is a significant cluster in the right temporal parietal junction, with relaxometry times up to 3.40 SD above controls (peak relaxometry value = 65.5 ms). Lastly, the right superior cerebellum contains a significant cluster with relaxometry times up to 8.72 SD above controls (peak relaxometry value = 89.4 ms).

**Case 8**

Case study eight suffered an mTBI during a rugby game, during which he lost consciousness momentarily. No additional clinical data was available for this patient regarding specific symptom complaints during or post-injury. BIST scores indicate a rating of 8/10 for the statement “I don’t like bright lights” and a total initial score of 52/160. This patient has experienced two previous mTBIs, with the last one two months prior in 2023. Recovery time for the current injury is recorded as 27 days. Upon examination of T2 relaxometry images, two significant findings were discovered. The first is in the cingulate cortex, with relaxometry times up to 8.27 SD higher than controls (peak relaxometry value = 73.5 ms) and the second overlaps the left superior Wernicke’s area and inferior supramarginal gyrus, with relaxometry times up to 16.31 SD (peak relaxometry value = 76.1 ms).

**Case 9**

Case study nine suffered an mTBI during a rugby game when he fell and hit the back of his head. The patient reported having double vision at the time of injury and then developing brain fog, slowed thinking and indecisiveness three days post-injury. At the time of his clinical assessment, 12 days post-injury, the patient reports no symptoms and a 0/10 rating for all BIST statements. No available data indicates whether the patient has suffered any previous mTBIs. Recovery time for this patient is recorded as 18 days. When looking at the T2 relaxometry images, two significant voxel clusters are found. The first is in the left superior parietal lobe, with relaxometry times of up to 8.81 SD higher than controls (peak relaxometry value = 65.7 ms), and the second is in the right posterior insula, with relaxometry times up to 5.31 SD higher than controls (peak relaxometry value = 70.3 ms).

**Case 10**

Case study 10 sustained an mTBI while surfing. This patient reports experiencing a seizure and loss of consciousness with this mTBI. No additional clinical data was available for this patient regarding specific symptom complaints during or post-injury. The patient indicated a 7/10 BIST rating for the statement “I don’t like bright lights”, a 7/10 for “I don’t like loud noises”, a 6/10 for “I feel dizzy or like I could be sick”, an 8/10 for “I feel clumsy”, a 7/10 for “It takes me longer to think”, a 7/10 for “I forget things”, and a 7/10 for “I get confused easily”, with a total initial BIST score of 69/160. The patient reports numerous previous mTBIs, with the exact amount and timing of the most recent one being unknown. The participant did not report the recovery time for the current mTBI. Three significant voxel clusters were located when examining the T2 relaxometry images. The first was a large cluster in the left superior parietal lobe, with relaxometry times up to 15.92 SD higher than controls (peak relaxometry value = 54.9 ms). The second was another large cluster, in the right inferior parietal lobe, with relaxometry times up to 14.48 SD above controls (peak relaxometry value = 66.0 ms) and the last was in the right superior parietal lobe, with relaxometry times up to 8.46 SD above controls (peak relaxometry value = 70.8 ms).

**Case 11**

Case study 11 suffered an mTBI in rugby when another player’s shoulder hit the right side of his head. At the time of the injury, the patient complained of feeling dizzy, dazed, with a headache and neck pain. At clinical assessment, 11 days post-injury, he indicated sensitivity to light and feeling “foggy” with mild cognitive symptoms. BIST scores were low with no symptom domains or individual statements obtaining abnormally high scores and an initial total score of 18/160. This patient has suffered four previous mTBIs, with the most recent being in November 2021. The recovery time for the current mTBI is recorded as 24 days. Three significant voxel clusters are apparent when assessing the T2 relaxometry MR images. The first is in the left parahippocampal cortex, with relaxometry times up to 10.37 SD higher than controls (peak relaxometry value = 76.5 ms). Secondly, there is a significant cluster in the left orbitofrontal cortex, with relaxometry times up to 4.83 SD higher than controls (peak relaxometry value = 6.5 ms). Next, there is a cluster in the right anterior occipital lobe, with relaxometry times up to 10.22 SD higher than controls (peak relaxometry value = 79.2 ms). Lastly, there is a significant cluster in the right orbitofrontal cortex, with relaxometry times up to 4.34 SD higher than controls (peak relaxometry value = 74.7 ms).

**Case 12**

Case study 12 suffered an mTBI during a rugby tackle, with the left side of his head hitting the ground and the knee or elbow of another player hitting the right side of his head. At the time of injury, he lost consciousness for a few seconds, was reported to be “zoning out” and experiencing headaches. At his clinical assessment, 11 days post-injury, he reports having some difficulty concentrating at school, as well as having headaches and some pressure in his head. BIST scores indicate a rating of 8/10 for the statement “I don’t like bright lights” and a 9/10 for the statement “I don’t like loud noises”, with a total initial BIST score of 61/160. This patient has not experienced any previous mTBIs and recovery time is recorded as 23 days. Two significant voxel clusters are apparent when assessing the T2 relaxometry MR images. These are in the right superior cerebellum, with a relaxometry time up to 13.15 SD higher than the control group average (peak relaxometry value = 79.5 ms) and the right superior temporal sulcus, with relaxometry times up to 10.23 SD higher than controls (peak relaxometry value = 69.5 ms).

**Case 13**

Case study 13 sustained two separate head injuries during a rugby game; the first when his head collided with another player’s knee, and then the second when he received an elbow to the left side of his face. The patient reports feeling shocked and dazed at the time of the injuries and reports feeling pressure in his head as well some neck pain, fogginess and disturbance to cognitive functions such as memory four days post-injury. There was no data available for this patient with regards to whether loss of consciousness occurred or whether any previous mTBIs have been suffered. BIST scores were low with no symptom domains or individual statements obtaining abnormally high scores, and an initial total score of 42/160. Recovery time for this patient is recorded as 17 days. One significant voxel cluster was identified when evaluating this patient's T2 relaxometry MR images. This was in the left anterior temporal region, with relaxometry times up to 4.70 SD above controls (peak relaxometry value = 125.0 ms).

**Case 14**

Case study 14 suffered an mTBI during a tackle in a football game when the ball was kicked directly at face, losing vision for a few seconds. No additional clinical data was available for this patient regarding specific symptom complaints during or post-injury. BIST scores indicate a 6/10 rating for the statement “I don’t like bright lights” but low scores for other statements with an initial total score of 61/160. This patient has suffered one previous mTBI in 2018. Recovery time for the current mTBI is recorded as 20 days. Four significant voxel clusters were discovered when analysing the T2 relaxometry MR images. The first area overlaps the left superior Wernicke’s area and inferior supramarginal gyrus, with relaxometry times up to 25.44 SD higher than controls (peak relaxometry value = 70.2 ms). The next overlaps the left posterior corpus callosum and anterior occipital lobe, with relaxometry times up to 13.83 SD higher than controls (peak relaxometry value = 57.7 ms). In addition, there is a significant cluster in the left hippocampus, with relaxometry times up to 8.38 SD higher than controls (peak relaxometry value = 274.1 ms) and in the right premotor cortex, with relaxometry times up to 19.60 SD higher than controls (peak relaxometry value = 87.6 ms).

**Case 15**

Case study 15 was hit on the right side of his maxilla by an elbow during a rugby training session, resulting in an mTBI. The patient had ataxia (impaired coordination) and confusion directly following the incident. During his clinical assessment two days post-injury he reports a high symptom load as indicated by vision motion sensitivity testing, for example. However, the patient’s BIST scores were low with no symptom domains or individual statements obtaining abnormally high scores and an initial total score of 56/160. There is no available data for whether the patient has experienced any previous mTBIs and recovery time for this mTBI is recorded as 18 days. Four significant voxel clusters were found when analysing the T2 relaxometry MR images. The first is in the right inferior parietal region, with relaxometry times up to 12.33 SD higher than controls (peak relaxometry value = 70.6 ms). Next, there is a significant cluster in the right posterior parietal region, with relaxometry times up to 19.85 SD higher than controls (peak relaxometry value = 66.7 ms). Furthermore, there is a significant cluster that overlaps the right superior cerebellum and inferior occipital region, with relaxometry times up to 5.78 SD higher than controls (peak relaxometry values = 104.7 ms) and in the right medial prefrontal cortex, with relaxometry times up to 8.13 SD higher than controls (peak relaxometry value = 83.9 ms).

**Case 16**

Case study 16 suffered an mTBI during a rugby game when the knee of his opponent made contact with the back of his head. The patient experienced loss of balance at the time of injury and 24-hours-later developed numbness in his hands, blurry vision and headaches, with pins and needles in his hands, dizziness and headaches still at his clinical assessment 11 days post-injury. BIST scores indicate a 7/10 rating for the statement “I feel dizzy, or like I could be sick” and an initial total score of 54/160. Recovery time for this mTBI is recorded as 117 days. Furthermore, the patient has suffered four previous mTBIs, with the latest one occurring in 2021. When analysing the T2 relaxometry images, one significant voxel cluster was identified in right sensorimotor cortex, with relaxometry times up to 14.86 SD above controls (peak relaxometry value = 74.0 ms).

**Case 17**

Case study 17 suffered an mTBI during a tackle in a rugby game. No clinical notes are available for this patient in relation to his symptoms at the time of the injury or at the time of clinical presentation. BIST scores indicate a rating of 6/10 for the statement “I don’t like loud noises”, an 8/10 for “It takes me longer to think”, a 9/10 for “I forget things”, a 6/10 for “I get confused easily”, a 6/10 for “I have trouble concentrating”, a 6/10 for “I get angry or irritated easily”, a 7/10 for “I feel tired during the day” and an 8/10 for “I need to sleep a lot more or find it hard to sleep at night”, with an initial total BIST score of 79/160. This patient reports two previous mTBIs, with the most recent mTBI only two months prior in May 2023. Recovery time for the current mTBI is recorded as 66 days. Three significant voxel clusters were identified when analysing the T2 relaxometry MR images. Firstly, in the cingulate cortex with relaxometry times up to 11.08 SD above controls (peak relaxometry value = 99.0 ms), secondly in the left superior frontal lobe with relaxometry times up to 6.05 SD above controls (peak relaxometry value = 259.4 ms) and thirdly in the left posterior cerebellum, with relaxometry times up to 5.37 SD above controls (peak relaxometry value = 1.6e+08 ms).

**Case 18**

Case study 18 suffered an mTBI during a rugby game when another player’s knee made contact with his head and he lost consciousness momentarily. No additional clinical data is available for this patient regarding specific symptom complaints during or post-injury. BIST scores were low with no symptom domains or individual statements obtaining abnormally high scores and an initial total score of 2/160. This patient reports no previous mTBI and the recovery time for the current injury is recorded as 26 days. The radiologist report indicated a tiny focus of susceptibility in the left superior frontal gyrus (which could be vascular or represent a tiny focus of nonspecific haemosiderin deposition). When analysing T2 relaxometry MRI data for this patient, a significant cluster of voxels was identified in the left superior frontal lobe, with relaxometry times up to 5.28 SD higher than controls (peak relaxometry value = 303.7 ms).

**Case 19**

Case study 19 was elbowed in the face during a futsal game and suffered an mTBI as a result. No additional clinical data is available for this patient regarding specific symptom complaints during or post-injury. BIST scores indicate a high rating for 12 different statements, with an initial total score of 117/160. The patient reported an 8/10 for the statement “I don’t like bright lights”, a 9/10 for “I don’t like loud noises”, a 7/10 for “I feel dizzy or like I could be sick”, a 10/10 for “If I close my eyes, I feel like I am at sea”, a 10/10 for “It takes me longer to think”, a 9/10 for “I forget things”, a 9/10 for “I get confused easily”, a 9/10 for “I have trouble concentrating”, an 8/10 for “I get angry or irritated easily”, a 7/10 for “I feel restless”, an 8/10 for “I feel tired during the day”, and an 8/10 for “I need to sleep a lot more or find it hard to sleep at night”. The patient reports no previous mTBIs and the recovery time for the current injury is recorded as 32 days. No significant clusters of voxels were identified when analysing the T2 relaxometry images.

**Case 20**

Case study 20 sustained an mTBI while engaging in jiu-jitsu when he was hit with a knee to the head. There are no notes available pertaining to his symptoms at the time of injury, only mention of headaches at his clinical presentation 11 days post-injury. BIST scores were mostly low, however, there was a rating of 6/10 for the statement “I feel tired during the day” and 6/10 for “I need to sleep a lot more or find it hard to sleep at night”, with an initial total BIST score of 28/160. This patient reports two previous mTBIs, with the most recent being in 2012. The recovery time for the current mTBI is reported as 38 days. When analysing the T2 relaxometry images, seven significant voxel clusters were identified. One in the left anterior cingulate cortex, with relaxometry times of up to 17.51 SD above controls (peak relaxometry value = 66.3 ms), one in the left medial frontal lobe with relaxometry times up to 5.68 SD above controls (peak relaxometry value = 121.1 ms), one in the left sensorimotor cortex with relaxometry times up to 4.57 SD above controls (peak relaxometry value = 79.7 ms), one in the left precuneous of the parietal lobe with relaxometry times up to 5.49 SD above controls (peak relaxometry value = 39.0 ms) and one in the left temporoparietal junction with relaxometry times up to 5.60 SD above controls (peak relaxometry value = 100.3 ms). Furthermore, there was a significant cluster in the right anterior cingulate cortex with relaxometry times up to 15.48 SD higher than controls (peak relaxometry value = 73.5 ms) and lastly, in the right superior parietal lobe with relaxometry times up to 4.89 SD higher than controls (peak relaxometry value = 22.1 ms).

**Supplementary Material 3**

We simulated 3D images with Gaussian noise and 10000 voxels. We inserted a single ‘abnormality’ of connected voxels in one of the images (ground truth). We wanted to estimate *how many control samples we need to detect this abnormality* (i.e., a true positive finding). We used 3 different sizes of ‘abnormality’ (0.5% of image, 2.5% of image and 5% of image), with 5 different effect sizes. ‘Abnormal voxels’ have values 1, 2, 3, 4 and 5 standard deviations of the mean. As shown below, we start to consistently detect significant voxels (yellow indicates significant ‘abnormal’ voxels, *p* < 0.05, FDR corrected) an effect size of 3 standard deviations above the mean, using between 20-30 control samples. It is worth noting that the effect size was more important for detecting true positive voxels than the size of the abnormality.


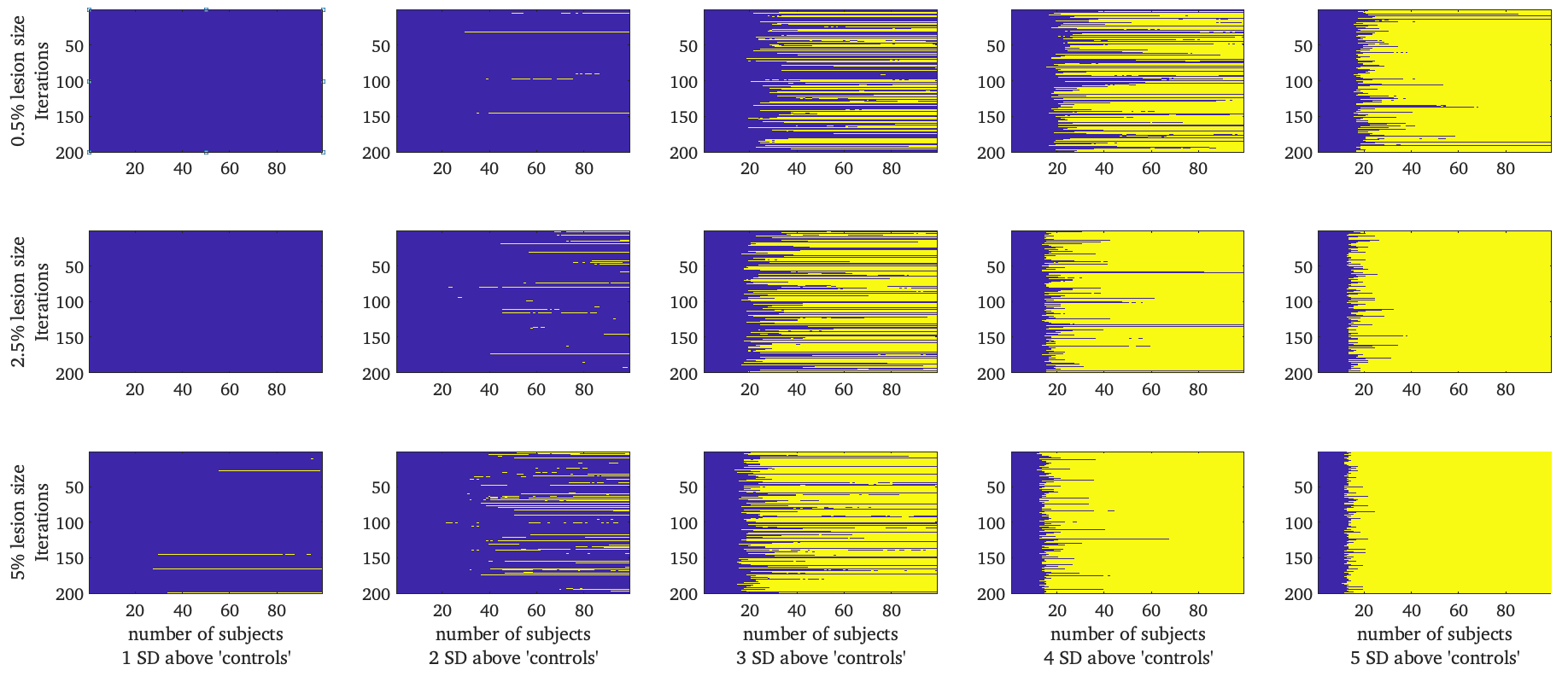

Supplement: Supplementary Data 1 [file mmc1.docx]
